# 177. Follow-up Respiratory Cultures in Suspected Ventilator-associated Pneumonia: An Opportunity for Diagnostic Stewardship

**DOI:** 10.1093/ofid/ofac492.255

**Published:** 2022-12-15

**Authors:** Cyndi Gonzalez Gomez, Owen Albin

**Affiliations:** University of Michigan Medical School, Pittsburgh, Pennsylvania; University of Michigan Medical School, Pittsburgh, Pennsylvania

## Abstract

**Background:**

Ventilator-associated pneumonia (VAP) is overdiagnosed in intensive care units (ICUs) and contributes to antibiotic overuse. Follow-up/test-of-cure culturing practices have garnered stewardship attention in urinary tract infections and gram-negative bacteremia but remain unexplored in suspected VAP. We aimed to ascertain the stewardship implications of repeat respiratory culturing practices in ICU patients.

**Methods:**

Retrospective descriptive cohort study of adult patients in Michigan Medicine ICUs requiring invasive mechanical ventilation in 2019 who had repeat respiratory cultures (RCx) performed within 72 hours after an index respiratory culture. Relevant patient demographics, comorbidities, culture indications and consequent antimicrobial modifications based on RCx were captured.

**Results:**

Of 2340 total respiratory cultures performed, 286 (12%) were RCx. Patient characteristics and indications for culture collection are shown in Figures 1 and 2, respectively. Only 12 patients (4%) had antimicrobial agents modified based on growth of a pathogenic organism from a RCx not covered by the prior antimicrobial regimen. In cases where RCx grew an identical organism with an identical susceptibility profile to the initial respiratory culture, existing antibiotic treatment durations were extended for microbiologic positivity in 12% of cases. Notably, 94 patients (33%) had RCx performed for persistent fevers or leukocytosis, without corresponding changes in ventilator requirements, changes in endotracheal secretions or worsening appearance on chest imaging.

**Figure:**
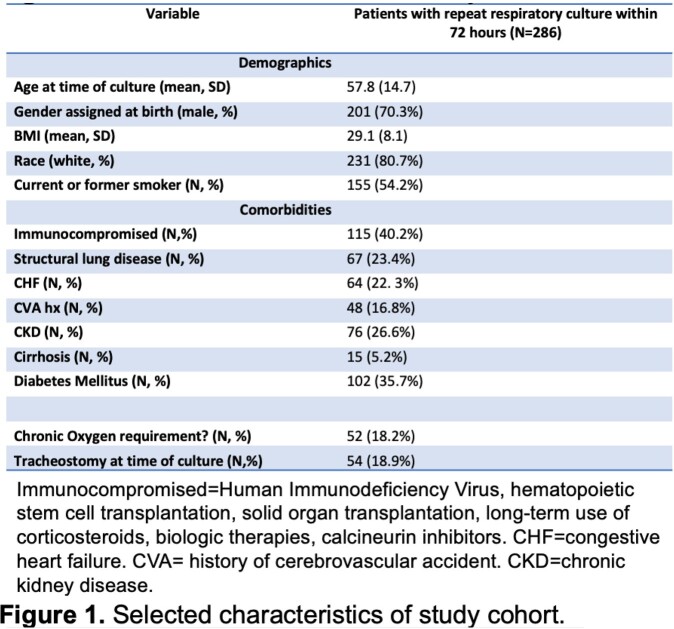

**Figure:**
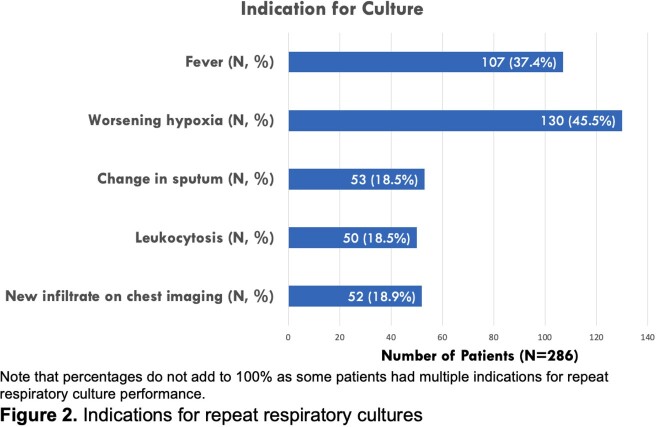

**Conclusion:**

RCx performed within 72 hours of an initial respiratory culture in mechanically-ventilated patients was both common and low-yield. RCx not uncommonly precipitated unnecessary prolongation in antimicrobial treatment durations, and nearly one-third of patients had RCx obtained for persistent fevers or leukocytosis without other signs of VAP treatment failure, a suspect indication for culture collection given that the median time to clinical improvement in VAP clinical trials in 4-5 days. Limiting repeat respiratory cultures in ICU patients represents a potentially valuable opportunity for diagnostic stewardship.

**Disclosures:**

**Owen Albin, MD**, Charles River Laboratory: Advisor/Consultant|Cipla Pharmaceuticals: Advisor/Consultant|Shionogi Inc: Advisor/Consultant.

